# Recent advances in personalizing cardiac arrest resuscitation

**DOI:** 10.12688/f1000research.17554.1

**Published:** 2019-06-21

**Authors:** Cyrus E. Kuschner, Lance B. Becker

**Affiliations:** 1 Donald and Barbara Zucker School of Medicine at Hofstra/Northwell, 500 Hofstra Boulevard, Hempstead, NY, 11549, USA

**Keywords:** Cardiac Arrest, Resuscitation, Metabolic Phase, Cardiopulmonary Resuscitation, CPR

## Abstract

Cardiac arrest remains a significant cause of death and disability throughout the world. However, as our understanding of cardiac arrest and resuscitation physiology has developed, new technologies are fundamentally altering our potential to improve survival and neurologic sequela. Some advances are relatively simple, requiring only alterations in current basic life support measures or integration with pre-hospital organization, whereas others, such as extra-corporeal membrane oxygenation, require significant time and resource investments. When combined with consistent rescuer and patient-physiologic monitoring, these innovations allow an unprecedented capacity to personalize cardiac arrest resuscitation to patient-specific pathophysiology. However, as more extensive options are established, it can be difficult for providers to incorporate novel resuscitation techniques into a cardiac arrest protocol which can fit a wide variety of cases with varying complexity. This article will explore recent advances in our understanding of cardiac arrest physiology and resuscitation sciences, with particular focus on the metabolic phase after significant ischemia has been induced. To this end, we establish a practical consideration for providers seeking to integrate novel advances in cardiac arrest resuscitation into daily practice.

## Advances in cardiac arrest outcomes

Cardiac arrest (CA) remains a significant cause of death and disability throughout the world; in the United States of America (US), approximately 395,000 cases occur out-of-hospital (OHCA) and 200,000 cases occur in-hospital (IHCA) per year ^1, 2^. This disease process carries a significant global impact; as such, a number of international registries have emerged across regions such as Asia, Australia, and Europe in an attempt to understand the disease impact and progress toward improved outcomes ^3– 5^. Both IHCA and OHCA carry significant risks of morbidity and mortality. However, OHCAs hold significantly worse outcomes, ostensibly due to longer periods of delay until receiving cardiopulmonary resuscitation (CPR), increasing the risk of arrests with cardiac origin progressing to a non-shockable rhythm and allowing prolonged periods of either no- or low-flow circulation to cause systemic organ damage. After prolonged periods of CA without return of spontaneous circulation (ROSC), outcomes worsen significantly and carry drastically reduced efficacy of CPR.

Despite advances in our understanding of CA pathophysiology, mortality and neurologic morbidity remain poor. Sasson *et al*. shows that OHCA survival in the US remained stable at approximately 5.5% between 1982 and 2012 ^6^. Recent trends show an improvement in OHCA survival across the US, corresponding to an increase in initiation of bystander CPR at home or in public as well as first-responder defibrillation at home ^7^. Indeed, the primary variables associated with recent differences in the US OHCA outcomes appear to be initiation of bystander CPR and time until responder defibrillation ^8^. However, Girotra *et al*. present evidence that this may have been due to a shift in the demographics of patients with CA as more recent IHCA cases consist of significantly younger patients and a decreased incidence of IHCA due to heart failure, previous myocardial infarction, and presentation with a shockable rhythm ^9^. As such, it remains uncertain whether recent improved outcomes are directly caused by changes in clinical practice or are due to a shifting epidemiologic current toward pathophysiology susceptible to rapid defibrillation.

In an attempt to improve CA outcomes, a wide variety of resuscitation techniques have been studied. Some are relatively simple, requiring only alterations in current basic life support measures, whereas others require significant time and resource investments. Each of these interventions allows further personalization of the CA resuscitation, providing extensive options to the provider. However, arresting individuals come with varying degrees of complexity. A witnessed IHCA with a shockable rhythm provides a completely different pathophysiology than an unwitnessed OHCA with 25 minutes of attempted resuscitation. As such, it can be difficult to incorporate novel resuscitation techniques into a CA protocol which can fit both simple and complex cases.

## Personalizing cardiac arrest resuscitation through the hierarchical model

As our understanding of CA and resuscitation physiology has improved, new technologies are fundamentally altering our potential to improve survival during CAs. These advances allow a modernized approach to resuscitation through the “hierarchical model”, in which patients are provided progressively more advanced resuscitation technologies in a logical stepwise manner. Resuscitations begin at the bottom of the model with the simplest and most universally applicable therapies before progressing toward the top of the hierarchy with individualized advanced therapies. In the setting of CA, the hierarchical model starts with simple hands-only CPR/basic life support care before advancing to monitoring the quality (rescuer protocol adherence) of rescuer actions by assessing factors such as compression, ventilation, drugs, and other advanced cardiac life support therapies. Higher on the hierarchy is monitoring the direct impact of resuscitation actions on the individual patient’s physiology through monitoring factors such as CO _2_ generation, O _2_ consumption, arterial and venous blood flow, and cerebral perfusion. Ultimately, further advanced resuscitation options can be chosen on the basis of the physiologic needs. In this way, the hierarchical model facilitates initial universal actions before allowing CA resuscitations to be progressively personalized to the needs of the individual patient. This article will fit recent advances in our understanding of CA physiology and resuscitation sciences into a practical consideration for providers.

## Cardiac arrest is a time-sensitive disease

**Figure 1.**
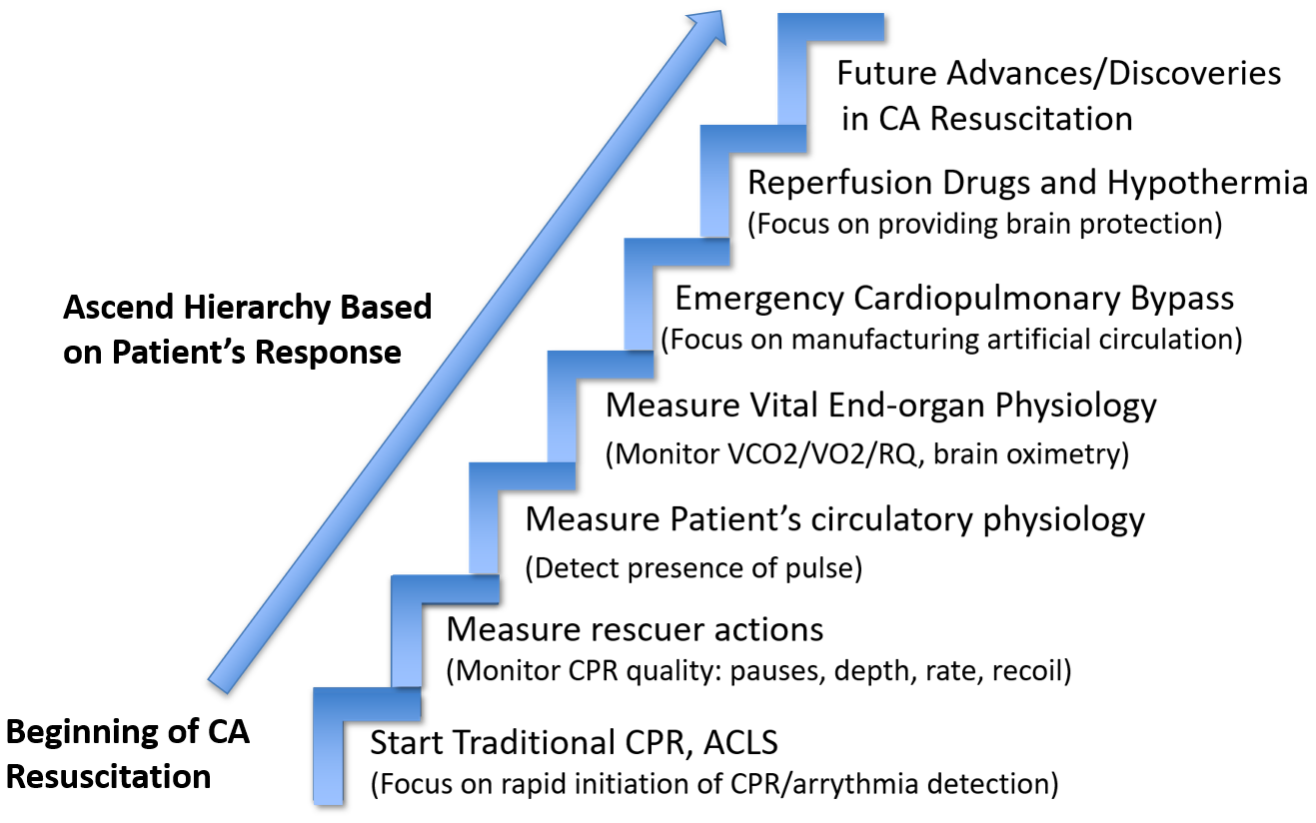
Hierarchical model of modern resuscitation. ACLS, advanced cardiac life support; CA, cardiac arrest; CPR, cardiopulmonary resuscitation; RQ, respiratory quotient; VCO _2_, ventilatory carbon dioxide; VO _2_, ventilatory oxygen.

**Figure 2.**
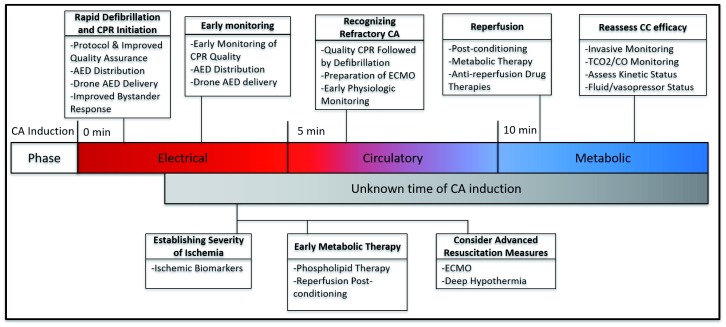
Contextualizing novel cardiac arrest interventions on the basis of phase of cardiac arrest. AED, automated external defibrillation; CA, cardiac arrest; CPR, cardiopulmonary resuscitation; ECMO, extra-corporeal mechanical oxygenation; TCO _2_/CO, total carbon dioxide/cardiac output.

Immediately upon CA induction, the most beneficial intervention is the rapid application of CPR with potential defibrillation of any potential arrhythmia ( Figure 1). To this end, movements have been made to decrease time until ROSC by improving emergency medical services (EMS) response time, CPR training in populations, and both number and display of automated external defibrillations (AEDs) in communities throughout the US. Indeed, focusing on early CPR initiation by bystanders has been recognized as the most modifiable factor for CA survival, emphasizing the importance of early resuscitation in providing perfusion prior to prolonged ischemic damage ^10^. Ultimately, these measures led to an improvement in OHCA survival despite its continued poor overall survival and neurologic sequela ^11^. However, significant strides remain essential to expedite arrhythmia detection and treatment ( Figure 2). Recently, the novel use of unmanned aerial drones to deliver AEDs to reported OHCAs was reported by Pulver *et al*., who demonstrated significant improvement in OHCA rescuer speed of AED access, increasing AED delivery within 1 minute by 76.5% compared with traditional EMS delivery of AEDs ^12^. Another emerging tool connects CPR-trained individuals through a cell phone application, notifying members when an OHCA has occurred in their vicinity. Theoretically, the use of applications such as PulsePoint (US), Heart Runner (Sweden), or GoodSAM (UK) could decrease time until initial CPR application; however, further research is necessary to assess their impact on OHCA outcomes ^13^.

## Assessing and ensuring quality cardiopulmonary resuscitation

Despite well-established protocols, providing consistent CPR remains difficult. Previous literature demonstrated significant discrepancies between established CPR guidelines and the practice of CPR in IHCA and OHCA patients in the early 2000s ^14, 15^. Later findings demonstrated that maintaining a consistent waveform in accordance with guidelines continued to be difficult despite updates in 2013 ^16^ and 2015 ^17^. Ultimately, this can lead to variance in compression rates ^18^, length of compression pauses, compression depth ^19^, and ventilation rates ^20^. These markers of poor CPR quality are frequently found during rescuer exhaustion, leading to diminished quality of reperfusion and ventilation over time. Feedback mechanisms offer an emerging mechanism to provide real-time quality assurance for rescuers ( Figure 1 and Figure 2). Real-time feedback can take many forms, tracking factors such as compression characteristics ^21^ or end-tidal carbon dioxide (EtCO _2_) output, ideally allowing practitioners to intuitively recognize and fix failing resuscitation techniques.

## Optimizing cardiopulmonary resuscitation, the evolution of an ideal waveform

Just as rescuer-focused real-time feedback improves rescuers’ capacity to maintain consistent protocol adherence, patient physiology–focused feedback allows practitioners to flexibly alter the protocol to match the individualized needs of the CA patient ( Figure 1). Protocol for CPR via chest compressions (CC) focuses on the use of a CC pattern to artificially establish circulation and, when combined with ventilation, allows reoxygenation via gas exchange. As such, it is essential that the primary purpose of CC is to establish forward blood flow. Compressions can be characterized as a “waveform” pattern, with unique variables such as compression rate, velocity, depth, and hold time as well as release velocity, time, and pause length. Current protocol describes the ideal waveform as a compression to a depth of at least 2 inches and less than 2.4 inches with a rate of at least 100 CC per minute and no more than 120 CC per minute, allowing complete compression release ^22^. Although this provides a clinically relevant protocol which can be reliably performed manually, the exclusion of other waveform characteristics allows variability in CPR performance. For example, recent literature has reported discrepancies in the clinical significance of compression release parameters. In two analyses using CPR defibrillator acceleration monitors, Cheskes *et al*. ^23^ found no impact of release velocity on survival whereas Kovacs *et al*. ^24^ found a survival benefit associated with faster release times. Using a swine model to monitor vascular flow during mechanical CC, Lampe *et al*. found that although longer release times of 300 ms were detrimental to ensuring blood flow, there was no difference in hemodynamic characteristics between release times of 200 and 100 ms ^25^. Instead, 100 ms release times were associated with increased endotracheal CO _2_ production. This suggests that variance in compression release time may play a significant role towards ensuring effective ventilation than blood flow.

## Does a single ideal waveform exist

Inherent to in the current CC protocol, which is designed to optimize blood flow, is the implicit assumption that a single compression waveform can sustain perfusion equally for all patients throughout the process of CPR. However, as resuscitation efforts are performed, the underlying anatomy and physiology of arresting individuals change. Broken ribs change the effect of compressions on inducing forward blood flow, the heart changes from a pump to a reverse pressure-driven system, and redistribution of plasma volume to the interstitium as edema, mesenteric vasculature through pooling, and venule system through loss of arteriole tone. Ultimately, this multi-system redistribution induces a hypotensive state which decreases right atrial filling capacity ^26^. Can a single CC waveform maintain the same efficacy through the dynamic changes in CA physiology? Using a swine model to simulate ventricular fibrillation (VF)-induced CA, Lampe *et al*. used a programmable mechanical CC device to assess the impact of cycling a variety of 2-minute CPR waveforms over the course of 50 compression minutes ^26^. Physiologic monitors alongside flow probes at arterial (common carotid, aorta, and abdominal aorta) as well as venous (external jugular, right atrial, and inferior vena cava) sites were used to assess how physiologic parameters change through CA resuscitation as well as the efficacy of difference waveforms in optimizing blood flow over time. Over the course of five CPR cycles, mean arterial pressure and right atrial pressure decreased from 30 and 17 mm Hg to approximately 14 mm Hg. This development of hypotension is consistent with the third-spacing of intra-vascular volume. Furthermore, the ideal waveform changed as a function of time, favoring compression patterns with a lower rate and shorter duty cycle. This demonstrates that, over time, the primary variable which impacts CA blood pressure is filling time, favoring a slower waveform. As such, the current literature does not support a single optimal waveform to sustain forward blood flow and ventilation, suggesting that resuscitation must be optimized.

Theoretically, the time-dependent hemodynamics associated with CA could be improved by initiating a dynamic approach to resuscitation. While fluids and vasopressors may provide some benefit during the early stages of resuscitation, their potential as tools to ameliorate hypoperfusion may be best found during the later stages of resuscitation to combat the effects of third-spacing. Similarly, a protocol which favors a compression rate of more than 100 CC per minute in the beginning of resuscitation and a slower compression rate over time could aid in ensuring adequate fill time during resuscitation. However, the hemodynamics of an arresting patient inherently change as each intervention is initiated. As such, blood pressure should ideally be dynamically monitored to assess the impact of resuscitation measures on blood pressure improvements. This can take the form of invasive monitoring such as direct A-line placement or central line assessment of central venous pressures. Alternatively, less invasive options such as peripheral limb blood pressure monitor, EtCO _2_, or trans-esophageal echocardiography ^27, 28^ can be used to assess peripheral limb pressure, ventilation, and cardiac output, respectively. Using these metrics, the provider can recognize the changing kinetic profile and tailor medical therapies or compression waveforms as needed. It can be difficult for providers to dynamically change manually applied CC waveforms during resuscitations. This opens further potential for electronic CC devices, which could be programmed to change the compression parameters in response to provider input.

## Improving quality defibrillation

Currently, guidelines recommend the use of defibrillation after 2 minutes of CPR if a “shockable” rhythm is present, requiring a pause in CPR to assess for rhythm. The likelihood that defibrillation will terminate VF corresponds predominately to the length of untreated VF as well as the degree of coronary perfusion increased by CPR, as the heart requires a return toward physiologic conditions with amelioration of metabolic dysfunction before a shock can return it to physiologic function. Using an amplitude-spectral area analysis to predict the likelihood of ROSC, a recent study demonstrated that this prediction model can significantly decrease the number of unsuccessful defibrillations and decrease length of time without CPR while assessing rhythm ^29^. Other avenues include the use of double sequential defibrillation, which maintains an uncertain role in CA resuscitation ^30^. Further work toward improving defibrillation quality may decrease peri-resuscitation morbidity and further tailor resuscitation techniques to the physiological status of the heart.

## Emerging new approaches to cardiac arrest resuscitation

After traditional CPR has failed to ensure ROSC, advancing up the hierarchical model focuses on more technologically advanced methods of returning perfusion. In particular, the development of veno-arterial extra-corporeal mechanical oxygenation (ECMO) has recently emerged as a promising intervention for CA. Recent meta-analyses have demonstrated that the current state of the literature on ECMO in CA is complicated by predominately single-institution, non-randomized studies with variable inclusion criteria and reported metrics of CA severity or recovery ^31^. However, in a meta-analysis of nine cohorts, Ouweneel *et al*. demonstrate that the use of ECMO in CA could improve long-term survival and neurologic outcomes by 15% and 11%, respectively, when compared with standard CPR ^32^. These findings were corroborated by a secondary analysis of studies with propensity-matched scoring, accommodating for the fact that patients selected for CA-ECMO were more likely to be young, male, with an underlying acute myocardial infarction requiring percutaneous intervention. The recent CHEER trial further corroborates the potential of ECMO in CA, with 54% of patients surviving to discharge with full neurologic recovery ^33^. Interestingly, survival was significantly associated with decreased time from collapse to initiation of ECMO (40 versus 78 minutes, *P* = 0.02) and perfusion team arrival to initiation of ECMO (16 versus 30 minutes, *P* = 0.01). Observationally, this corroborates the perspective that ECMO is most efficacious when initiated as soon as the CA is recognized as refractory to CPR after 10 to 15 minutes ^34^.

These results emphasize the importance of limiting the ischemic burden prior to ECMO initiation; however, they may inadvertently perpetuate the sole development of CA therapies for patients with the greatest likelihood of positive outcomes. Namely, patients with IHCA or witnessed OHCA with rapid CPR initiation. Patients with ischemic burden greater than 10 minutes represent a complex and severely at-risk population.

## The metabolic phase of cardiac arrest: highly lethal

After periods of long ischemia, patients have progressed past the initial “electrical” and “circulatory” phases of CA proposed by Weisfeldt and Becker in 2002 ( Figure 2) ^35^. At this point, the rescuer is placed in a complicated scenario, where the initiation of immediate CPR and ECMO re-introduce the metabolically deranged body to near-physiologic levels of oxygen, inducing reperfusion injury. Importantly, the rapid initiation of first-responder CPR during initial ischemic damage of the electrical phase can drastically improve survival. This highlights the essential component of population education and empowerment to begin resuscitation, potentially through compression-only CPR. However, many providers are faced with resuscitation after prolonged ischemia has placed the patient at risk of reperfusion injury. This process constitutes a complex shift in cellular metabolic activity due to the lack of oxygen which has been reviewed in detail elsewhere ^36^. In brief, the lack of ATP generation due to ischemia causes an intracellular redoxshift which, without sufficient oxygen to facilitate complex IV reduction, causes electrons to react with remaining oxygen, resulting in reactive oxygen species (ROS) formation. Concurrently, diminished ATP generation stops the cell from maintaining electrolyte “upkeep”, causing calcium, sodium, and potassium shifts with secondary osmotic swelling of the mitochondria. When normal CPR is initiated and the body is returned to 25 to 40% of its normal circulation, near-physiologic levels of oxygen induce ROS generation, predominately by the oxidative phosphorylation (OXPHOS) proteins of the mitochondria. As such, ideal resuscitation would theoretically be targeted to the individual patient’s stage of metabolic dysfunction. Although recent advances provide new possibilities for personalized care, this remains an area of intense ongoing research.

## Treating the metabolic phase of cardiac arrest

One approach to limiting the impact of reperfusion injury in the patient with severe ischemia is the use of postconditioning (PC). By introducing brief pauses in CPR, tissues are exposed to less quantities of oxygen. Thus, by matching the diminished oxygen utilization of metabolically shocked tissues, PC theoretically weans tissues back to normal oxygen consumption capacity, limiting reperfusion injury while minimizing the production of ROS in metabolically deranged cells. The potential for PC was demonstrated by Segal *et al*., using a swine-VF model to compare 15 minutes of untreated VF followed by CPR versus CPR with four 20-second pauses during the first 3 minutes of CPR (CPR+PC) ^37^. Overall CPR+PC resulted in significant improvement in left ventricular ejection fraction at 1 and 4 hours, alongside improved neurologic function by cerebral performance category scoring at 24 hours ^37^.

Furthermore, in a recent study, Matsuura *et al*. investigate whether decreased brain and heart mitochondrial function post-resuscitation is predominately caused by abrupt reperfusion and thus could be improved by PC ^38^. Using a swine-VF CA model, four groups were established for this study: non-ischemic, 19-minute VF CA without CPR, 15-minute VF CA with 4-minute CPR, and 15-minute VF CA with 4-minute ischemic PC CPR. Ultimately, CPR with PC was associated with significant improvements in heart mitochondria respiratory capacity; however, no improvements in brain mitochondrial function were observed. Solely addressing ischemia and reperfusion does not appear to be enough to effectively improve neurologic sequela in CA. As such, this challenging area remains a prime target for ongoing research.

## Multiple concurrent solutions to the complex metabolic patient

Neurologic outcomes are typically poor in patients with unwitnessed OHCA. Any attempt to improve neurologic sequela in this patient population is complicated by the brain’s unique susceptibility to long-term ischemia. While limiting reperfusion injury can assist in ameliorating multi-organ damage, it alone appears to be unable to return the severely damaged brain to normal physiology. Recent literature supports the theory that post-ischemic phospholipid (PPL) dysfunction plays an integral role in this process ^39– 41^. Multiple pathways have been proposed, ranging from direct damage to membrane integrity, deleterious effects of increased lysophospholipids (LPLs) and free fatty acids, as well as the depletion of PPL reserves essential for continued metabolic function. Understanding this pathophysiologic process of metabolic dysregulation in brain tissue, including the explicit targeting of lipid metabolism, may be essential to establish the ideal resuscitation plan for improving neurologic sequela.

## Alterations in brain lipids during cardiac arrest

Alteration in brain lipids appears to be a critical event to the CA patient. The PPL profile of brain tissue includes phosphatidylethanolamine (58.3%), phosphatidylcholine (27.6%), phosphatidylserine (17.3%), phosphatidylinositol (2.9%), cardiolipin (0.5%), and phosphatidylglycerol (0.05%) ^39^. Much interest has been focused on PPLs as they represent the primary component of cell membranes and contribute to a wide variety of physiologic functions, including cellular signaling, transport, and metabolism. Ischemia and the introduction of ROS can dysregulate normal PPL function, increasing the concentration of polyunsaturated fatty acids (PUFAs) and cleaving PPLs, leading to increased LPL concentrations. This combination ultimately leads to increased concentrations of PUFAs such as arachidonic acid, which initiate pro-inflammatory signaling.

How then is PPL dysfunction unique to neuronal pathophysiology in the arresting patient? Physiologically, the brain carries a unique whole cell and mitochondrial PPL composition ^42^. Using a severe ischemic rat model with 30 minutes of asphyxiation followed by 60 minutes of cardiopulmonary bypass to simulate severe ischemia and reperfusion through ECMO, our group correlated PPL content in four major organs: brain, heart, kidney, and liver ^39^. Overall, after severe ischemia, all four organs had significant elevation in LPLs, demonstrating the increase in PPL derivatives caused by ischemia-induced breakdown of PPLs. However, whereas the heart, liver, and kidney were able to return their intracellular concentrations of LPLs to physiologic levels after 60 minutes of bypass, the brain was unable to normalize LPLs following reperfusion. Whether this is due to neuronal metabolic dysfunction with diminished PPL reservoirs or susceptibility to the pro-inflammatory effects of LPLs remains to be seen. Nevertheless, recent literature has further demonstrated the key role that neuronal metabolic dysfunction plays in CA pathophysiology. After ischemia and reperfusion, the brain’s PPL profile experiences a unique decrease in cardiolipin, a PPL key to normal mitochondrial physiology, leading to dampened complex I/III activity in neuronal electron transport chains ^43^. Furthermore, implementation of cardiolipin-targeted therapies through SS-31 has demonstrated improved survival and decreased metabolic dysregulation in animal models ^44^. How then could the practitioner translationally apply an understanding of these unique neuronal changes to try to improve neurologic function? If the post-ischemic brain is unable to replenish PPL components because of metabolic derangement, direct administration of PPL precursors or other mitochondrial stabilizing agents such as SS-31 could be pursued to aid the brain in replenishing normal PPL composition. Furthermore, as brain tissue is less able to moderate pro-inflammatory LPLs after reperfusion, administration of anti-inflammatory medications or hypothermia ^45^ may be beneficial in reducing pro-inflammatory neuronal damage. Finally, as the brain appears to be uniquely sensitive to ROS damage, administration of free-radical scavenging compounds could provide additional benefit to the brain.

## Post-resuscitation care: fine tuning neurovascular support

Factors to consider while managing CA patients after resuscitation include maintaining normocapnia, as intracranial pressure and cerebral blood flow are frequently affected by the long periods of hypoventilation associated with CA. Both hypocapnia and hypercapnia are frequently found after CA and are directly associated with adverse neurologic outcomes ^46^. Post-resuscitation maintenance of normocapnia has been associated with significant improvements in neurologic outcomes and should be emphasized during post-resuscitative care ^47^. Additionally, temperature control is essential during and after CA resuscitation. If allowed to maintain along the natural pathologic course of CA, patients will frequently develop hyperthermia, which is associated with poor outcomes. Although the ideal maintenance temperature is still being debated, current literature supports the maintenance of mild hypothermia to 33°C to 36°C. Thus, it is essential for clinicians to take direct measures to ensure that hyperthermia is avoided. As deep hypothermia is investigated in hypovolemic CA ^48^, its efficacy and potential for other etiologies will be worthwhile to assess.

## Personalizing cardiac arrest resuscitation

To illustrate the potential clinical execution of personalizing CA resuscitation, three potential cases will be discussed. Each demonstrates the initiation of a basic life support protocol alongside the use of additional measurements of physiologic conditions to tailor the escalation of resuscitation methods based on their responsiveness to interventions ( Figure 1 and Figure 2).

1. A 60-year-old man is observed to have a pulseless rhythm. CPR is initiated and after 2 minutes a VF is observed. Defibrillation is provided and ROSC obtained.
2. A 60-year-old man is brought to the hospital by ambulance. CPR had been initiated by bystanders and continued by EMS. While CC are continued, an arterial line is placed, demonstrating a low diastolic pressure. In response, the compression waveform is altered, increasing CPR rate, and there is an associated increase in diastolic pressure. Despite continued CPR, the patient’s pressure drops. Epinephrine is provided, increasing pressures and facilitating a return to a shockable rhythm. This patient is an epinephrine responder.
3. A 60-year-old man is brought to the hospital by ambulance. CPR had been initiated by bystanders and continued by EMS. While CC are continued, an arterial line is placed, demonstrating a low diastolic pressure. In response, the compression waveform is altered, increasing CPR rate, and there is an associated increase in diastolic pressure. Despite continued CPR, the patient’s pressure drops. Epinephrine is provided multiple times; however, consistent perfusion is unable to be obtained. This patient is an epinephrine non-responder. ECMO is initiated to maintain perfusion.

## Conclusions

Recent advances in understanding and treating CA present the practitioner with a wide variety of intervention options. These range from improving speed of initial basic life support measures to providing multi-disciplinary invasive interventions such as ECMO, with different patients requiring varying degrees of complex resuscitation techniques. Using the hierarchical model, practitioners can navigate emerging resuscitation techniques to maintain consistent quality despite the varying complexity of the patient’s disease burden. This process supports the personalization of CA resuscitation to aid in monitoring patient physiology such that interventions may be tailored to patient-specific pathophysiology. The field of resuscitation is at a point in time with great opportunities to improve resuscitation care across the full spectrum, from implementation to new discovery science. If we take advantage of these many opportunities, there is no question that survival rates will predictably improve as we move forward in the future.
